# Periodontitis and incident cognitive decline and dementia: A 15-year prospective cohort study of older men residing in Northern Ireland

**DOI:** 10.1177/13872877251401563

**Published:** 2025-12-12

**Authors:** Dominic N Farsi, Rena Abadalkareem, Gerry J Linden, Gareth J McKay, Claire T McEvoy, Michael McAlinden, Lewis Winning, Michael Hurley, Jo Kelly, Peter A Passmore, Clive Holmes, Chris C Patterson, Jessica L Teeling, Bernadette McGuinness

**Affiliations:** 1 Centre for Public Health, School of Medicine Dentistry and Biomedical Sciences, Queen's University Belfast, Belfast, UK; 2 Department of Nutritional Sciences, King's College London, London, UK; 3 School of Biological Sciences, Faculty of Environmental and Life Sciences, University of Southampton, Southampton, UK; 4 Northern Ireland Clinical Research Network, Belfast Health and Social Care Trust, Belfast, UK; 5 Dublin Dental University Hospital, Trinity College Dublin, Dublin, Ireland

**Keywords:** Alzheimer's disease, cognitive aging, cognitive decline, cognitive function, cognitive impairment, dementia, inflammation, periodontal disease

## Abstract

**Background:**

Periodontitis is a chronic bacterial infection that elicits systemic inflammation. While retrospective studies have linked periodontal pathogens with Alzheimer's disease (AD) and dementia, few have combined cognitive assessments, pathogen exposure, and inflammatory markers.

**Objective:**

To investigate the longitudinal risk between periodontitis, cognitive impairment and dementia.

**Methods:**

We examined the relationship between periodontitis and onset of mild cognitive impairment (MCI) and dementia over 15.6 years (SD 1.6) in older men from Northern Ireland enrolled in the PRIME-COG cohort, using logistic regression. We also assessed associations between exposure to periodontal pathogens and blood inflammatory markers.

**Results:**

Among 642 men, baseline periodontitis was not significantly associated with later onset of dementia and/or MCI (severe versus mild/none, OR 0.83, 95% CI 0.45–1.50, p = 0.923). However, having more teeth predicted lower risk (OR 0.95, 95% CI 0.91–0.99, p = 0.023). Dementia and/or MCI was associated with higher serum IL-6, IL-8, and IFN-γ at baseline, and IL-8 and TGF-β at follow-up. IgG levels to periodontal pathogens remained stable in men who developed dementia and/or MCI but declined in cognitively normal men. A positive correlation between IgG to periodontal pathogens and proinflammatory cytokines was observed in men who developed dementia and/or MCI.

**Conclusions:**

Clinical periodontitis was not associated with dementia or MCI onset, but tooth retention was protective. Elevated inflammatory markers in affected men suggest systemic inflammation may contribute to cognitive decline. Larger, more diverse cohort studies are needed to clarify the role of periodontal disease in dementia and AD risk.

## Introduction

Periodontitis is a chronic inflammatory disease that affects the gums and surrounding tissues. This disease results from a bacterial infection and is associated with several conditions that have a vascular and/or inflammatory component including stroke, coronary heart disease, rheumatoid arthritis, and diabetic complications.^1^ Severe periodontitis is the sixth most prevalent condition in the world; the global age standardized prevalence of severe periodontitis in 2010 was 11.2% rising steeply in those aged over 65 years.^2^

Alzheimer's disease (AD) is the most common type of dementia, representing 60–70% of all cases. AD prevalence increases with age from 5% in the seventh decade to 50% by the tenth decade of life.^3^ Sporadic late onset AD represents 98% of all AD cases and is likely due to a complex interaction of environmental, vascular, and genetic risk factors.^4,5^ In the context of environmental factors, the ‘Dementia prevention, intervention and care 2024 report’ by the Lancet Standing Commission identified 14 potentially modifiable risk factors for dementia prevention, which if addressed, could reduce dementia incidence by 45%,^6^ highlighting potential interventions targeting environmental and lifestyle factors.

It has become increasingly clear that inflammation has a key role in the onset and/or progression of AD. Convincing evidence for an inflammatory influence comes from genetic studies of patients with late onset AD that have implicated genes encoding proteins within immune and inflammatory response pathways.^7–14^ It has also been hypothesized that the *APOE* ε4 allele, an established risk factor for AD, modulates neuroinflammation and significantly increases carriers susceptibility to environmental associated risk factors.^15^ Our previous work has shown that individuals diagnosed with mild cognitive impairment (MCI) decline 5-fold faster when exposed to an acute systemic inflammatory event, such as a urinary tract infection. This accelerated decline in clinical symptoms is particularly evident in individuals with chronic low-grade inflammation, such as elevated serum levels of tumor necrosis factor alpha (TNF-α).^16^

Investigations of the periodontitis prevalence in older adults and the inflammatory milieu has demonstrated associations with increased risk of reduced cognitive functioning^17–21^ and dementia^22^ in cross-sectional studies. Further support from several retrospective population studies in 27,963 participants^23–26^ highlighted an increased risk of developing dementia in those with chronic periodontitis across a 1–10-year follow-up period. Other studies have shown that significant tooth loss predicts MCI,^24^ while alveolar bone loss has been associated with reduced cognitive score (Mini-Mental State Examination (MMSE) < 25) after adjustments for age, sex and education.^27^ Exposure to periodontal pathogens leads to activation of the immune system and elevated serum IgG and significantly higher periodontal pathogens levels in individuals that develop AD 10 years later.^28,29^ We demonstrated that individuals with mild to moderate AD decline significantly faster when co-existing periodontitis was present at baseline.^30^ This increased cognitive decline was associated with a relative increase in the pro-inflammatory state during the follow-up period, although serum IgG titers to *Porphyromonas gingivalis* were similar. These contrasting findings support recommendations for well-designed longitudinal studies to address the knowledge gap related to periodontitis as a dementia risk factor,^31^ further supported by a recent systematic review and meta-analysis that reported moderate quality of evidence and risk of bias amongst the identified studies.^32^

The role of periodontitis as a dementia risk factor has been hypothesized by several mechanisms: (1) Pro-inflammatory cytokines produced by periodontal tissues during periodontitis enter the bloodstream leading to AD-related neurological changes.^33,34^ Systemic inflammation may activate the already primed microglial cells within the central nervous system and hasten neurodegenerative processes.^35,36^ (2) Periodontopathogens such as *Porphyromonas gingivalis* (*Pg*), *Tannerella forsythia* (*Tf*), *Aggregatibacter actinomycetemcomitans* (*Aa*), and *Treponema denticola* (*Td*) or their membrane components (lipopolysaccharides (LPS)) may reach the brain via the systemic circulation and accelerate neuroinflammation perhaps through an increasingly permeable blood-brain barrier (BBB) associated with increasing age or the circum-ventricular organs not protected by the BBB.^37^ Td, Pg, and Tf have been isolated from the cerebral tissue of AD patients postmortem.^38–40^ (3) Periodontitis-induced cerebrovascular pathology, diabetic complications and cardiovascular disease could promote AD progression through a cascade of atherogenesis related systemic inflammation.^41,42^

The aim of present study is to investigate whether periodontal disease in 60–70-year-old men increases the risk of cognitive decline and the development of dementia after fifteen years follow-up and to investigate the role of inflammation in this process. Our *a priori* hypothesis was that diagnosis of periodontitis would be significantly associated with, and predict, incidence of both dementia and cognitive decline.

## Methods

### Study population

PRIME (Prospective Epidemiological Study of Myocardial Infarction) is a cohort study of cardiovascular disease in men from three centers in France and one in Northern Ireland (NI). During 1991–1994, 2748 men were recruited in NI which represented 5% of 50–60-year-old men in the greater Belfast area matching the local social class.^43^ In 2001–2003, the surviving participants were invited for re-screening and the collection of data on social circumstances, demographic background, tobacco use and alcohol consumption, and personal measures including weight and height. Fasting blood samples were obtained and analyzed for lipids and inflammatory markers. In addition, a clinical periodontal examination was completed for 1400 (69.7%) of the 2010 men who attended for re-screening,^44,45^ and subgingival plaque sampling was carried out on a subsample of participants. Baseline and follow-up cognitive assessments included MMSE.

Between 2018 and 2020, the 1400 men who had a clinical periodontal examination were invited to a further follow-up visit for the present study (PRIME-COG), where lifestyle information was collected as previously, along with further clinical and cognitive assessments. Inclusion criteria were restricted to participants who had previously undergone periodontal examination and dental plaque sampling at the previous visit. Edentulous participants were excluded to reduce effects from reverse causality, and those participants formally diagnosed with previous cognitive impairment or who scored < 24/30 on MMSE at baseline visit. A total of 642 participants were included in the present analysis as they attended visits in 2001–2003 and again in 2018–2020. Approval for the project was obtained from the Office for Research Ethics Committees (Northern Ireland); reference number 06/NIR02/107. Written informed consent was obtained from all participants following a detailed explanation of the study aims. The study was compliant with the Declaration of Helsinki guidelines and STrengthening the Reporting of OBservational studies in Epidemiology (STROBE) statement.^46^

### Periodontal examination

All periodontal examinations were completed by one of four dental hygienists who had completed a calibration assessment against a “gold standard” set by a senior clinical researcher prior to the study (GL). Regular monthly meetings took place to ensure inter- and intra-examiner consistency and reproducibility. Clinical periodontal measurements were made using a Michigan O periodontal probe (Hu-Friedy, Chicago, IL, USA) with William's markings. Measurements were made at the mesial, distal, buccal and palatal/lingual aspects of all teeth excluding third molars. Pocket probing depths were recorded as the distance from the gingival margin to the base of the clinical pocket with the probe tip parallel to the long axis of the tooth. Clinical attachment levels (CAL) were also recorded as the distance from the cement–enamel junction to the base of the clinical pocket. Measurements were made to the nearest millimeter and if doubt existed, the lower value was recorded. The number of teeth was also recorded, again excluding third molars. Periodontal disease was defined according to the Centre for Disease Control and the American Academy of Periodontology classification.^47^ “Severe periodontitis” required two or more interproximal sites with CAL ≥ 6 mm, not on the same tooth, and one or more interproximal sites with PD ≥ 5 mm. “Moderate periodontitis” was defined as two or more interproximal sites with CAL ≥ 4 mm, not on the same tooth, or two or more interproximal sites with PD ≥ 5 mm, not on the same tooth.

### Cognitive follow-up

In addition to repeating the baseline MMSE to assess change in cognition, a comprehensive assessment was carried out for the domains of attention/executive function, memory, language and visuospatial function using the Addenbrooke's Cognitive Examination Revised (ACE-R). Function was assessed using the Bristol Activities of Daily Living Scale (BADLS)^48^ and mood/depression assessed using the Geriatric Depression Scale (GDS) short form.^49^ Research nurses from the Northern Ireland Dementia Clinical Research Network were experienced in performing neuropsychological tests and venous blood collection. Serum was extracted, aliquoted and stored at −80°C for analysis. A comprehensive cognitive assessment included a consensus diagnosis of normal cognition for age, MCI, and/or dementia was made by three experienced clinical researchers (BMcG, PP, CH) who specialize in dementia/AD research using accepted clinical criteria.^50–52^

### Determination of serum IgG levels to dental bacterial pathogens, and serum inflammatory markers

Samples collected at baseline (PRIME) and follow-up visit (PRIME-COG) were assessed for serum immunoglobulin G (IgG) levels to four common bacterial periodontal pathogens; *Tf, Td, Aa,* and *Pg.* The full details are included in the Supplemental Material.

Pro-inflammatory cytokines including interferon gamma (INF-ɣ), interleukin (IL)-1β, IL-2, IL-4, IL-6, IL-8, IL-10, IL12p70, IL-13, TNF-α, were determined using an MSD^®^ multiplex assay (MSD Maryland, USA). CRP levels were determined using MSD^®^ single plex, whereas transforming growth factor beta (TGF-β1, TGF-β2, TGF-β3) were determined using U-plex MSD^®^ assays in line with recommended manufacturer's instructions.

### Model covariates

The following covariates were included in the multivariate models. Baseline age, *APOE* ε4 allele status (2 versus 1 versus 0 alleles), following *APOE* genotyping using Taqman Real Time PCR Assays (Thermo Fisher Scientific). Cardiovascular disease was characterized by previous myocardial infarction or an intervention such as angioplasty with stenting or coronary artery bypass. Cerebrovascular disease was recorded for participants who previously had a stroke. Diabetes and hypertension were self-reported and high cholesterol confirmed from blood samples during the 2001–2003 study visit. Lifetime smoking was expressed in five categories: never smoked, smoked other than cigarettes, smoked < 15 cigarette pack years, smoked ≥ 15 but < 30 cigarette pack years and smoked ≥ 30 cigarette pack years. Alcohol intake was also expressed as five categories of consumption: none, 1–128, 129–265, 266–461 and ≥ 462 mL/ week. Education level was segregated into ‘Primary’, ‘Secondary’, ‘Technical’, and ‘Higher’. Socioeconomic status was categorized into ‘High’, ‘Middle’, and ‘Low’ using a composite measure of material conditions based on the type of living accommodation (rented or owned, mortgaged), number of cars/vans/motorcycles in the household and the number of baths and/or showers and toilets in the home.^53^

### Statistical analysis

Prior to statistical analysis, data was assessed for normality by way of visual inspection of Q-Q plots and histograms and performing Shapiro-Wilk tests. The data for blood triglycerides were not normally distributed, therefore, log transformed values were used for analysis. For descriptive purposes, median and interquartile ranges are presented. Comparison of characteristics between men with dementia and/or MCI and those without, were compared using chi-squared tests for categorical variables and t-tests for continuous variables.

The relationship between periodontitis and dementia incidence later in life was assessed using logistic regression adjusted for possible confounders. Dementia incidence among study participants was small (4.8%) and therefore models were constructed with incidence of dementia and/or MCI as the outcome variable and periodontitis as the main predictor variable. For the models, periodontitis was categorized into 3 groups; ‘severe’, ‘moderate’, and ‘mild or none’, and comparisons were made between ‘severe’ versus ‘mild or none’ and ‘moderate’ versus ‘mild or none’. In addition to the primary outcome, our secondary outcome consisted of cognitive decline as measured by a fall of ≥ 3 points from MMSE score at PRIME-COG rescreening. The first model was constructed in a minimal facet, with periodontitis as the predictor variable for the cognitive endpoints. Following this, potential confounders were added sequentially to construct a fully adjusted model. The confounders included in the analysis were age, level of education, socioeconomic status, number of *APOE* ε4 risk alleles, cardiovascular disease (coronary heart disease, cerebrovascular disease), diabetes, hypertension, blood cholesterol level, number of teeth, smoking status and alcohol consumption. Odds ratios (OR) and 95% confidence intervals were calculated from the regression coefficients. Model performance was assessed using the Akaike information criterion (AIC) to determine the best performing model.

For the analysis determining associations between serum IgG levels to common periodontal pathogens and pro-inflammatory cytokines with cognitive outcomes, CRP and TGF-β were not normally distributed, therefore, log transformed values were used. Comparisons between participants with dementia and/or MCI and those who were cognitive healthy were performed using the Kruskal-Wallis test, followed by the Mann-Whitney U test for multiple comparison. For all analyses, two-tailed p values < 0.05 were considered significant. Statistical analyses were performed in R Studio^54^ SPSS 29.0 ((IBM, Armonk, NY) and Prism 10 (GraphPad software, San Diego, CA).

## Results

At the PRIME rescreening visit between 2001 and 2003, of the 642 participants included in the present analysis, 127 (19.8%) had severe periodontitis and 148 (23.1%) had moderate, while 367 (57.2%) men had either mild, or no sign of periodontitis. During the follow-up period from the PRIME rescreening visit (PRIME-COG; mean 15.6 (SD 1.6) years), 31 (4.8%) were diagnosed with dementia, 112 (17.4%) with MCI, and 37 (5.8%) experienced a reduction of ≥ 3 points in their MMSE score, indicative of cognitive decline. Summary characteristics for participants with and without dementia and/or MCI at PRIME-COG follow-up are compared in Table 1. Participants with dementia and/or MCI were older (p < 0.001), had less teeth (p = 0.010), higher serum levels of IL-8 (p = 0.047) and TGF-β (p = 0.022), a higher waist to hip ratio (p = 0.019), attained worse scores in MMSE (p < 0.001), BADLS (p < 0.001) and GDS (p < 0.001), were more likely to carry a greater number of *APOE* ε4 risk alleles (p < 0.001), have hypertension (p = 0.002), be educated to a lower level (p < 0.001) and be within a lower social class (p = 0.002), compared to participants without dementia and/or MCI.

**Table 1.** Comparison of characteristics of study population with and without dementia and/or MCI at PRIME-COG follow up (n = 642) ^a^.

Measure	Dementia / MCI		
	No (n = 500)	Yes (n = 143)	*p* ^b^
Age (y), mean (SD)	78.23 (2.76)	80.13 (2.83)	<0.001
Periodontal disease, n (%)			0.874
Severe	100 (20.0%)	27 (18.9%)	
Moderate	113 (22.6%)	35 (24.5%)	
Mild or none	286 (57.3%)	81 (56.6%)	
No. teeth, mean (SD)	21.23 (4.69)	19.26 (6.68)	0.010
Mini-Mental State Examination, mean (SD)	29.22 (0.96)	25.96 (2.03)	<0.001
Bristol Activities of Daily Living Scale, mean (SD)	1.20 (2.70)	1.48 (3.30)	<0.001
Geriatric Depression Scale, mean (SD)	1.59 (2.19)	1.78 (1.81)	<0.001
Diabetes, n (%)			0.049
Yes	4 (0.8%)	0 (0%)	
No	496 (99.2%)	143 (100%)	
CVD disease, n (%)			0.074
Yes	79 (15.8%)	32 (22.4%)	
No	421 (84.2%)	111 (77.6%)	
Hypertension, n (%)			0.002
Yes	136 (27.2%)	60 (42%)	
No	364 (72.8%)	83 (58%)	
High cholesterol, n (%)			0.115
Yes	170 (34%)	38 (26.6%)	
No	330 (66%)	105 (73.4%)	
*APOE* ε4, n (%)			<0.001
0	349 (69.8%)	86 (60.1%)	
1	97 (19.4%)	37 (25.9%)	
2	3 (0.6%)	8 (5.6%)	
Systolic blood pressure (mmHg), mean (SD)	136.69 (16.17)	139.48 (16.17)	0.030
Body mass index (kg/m2), mean (SD)	27.28 (2.98)	27.52 (2.87)	0.200
Waist to hip ratio (cm), mean (SD)	0.93 (0.06)	0.95 (0.06)	0.019
Total cholesterol (g/L), mean (SD)	5.56 (1.1)	5.38 (0.89)	0.603
Triglycerides (g/L), median [IQR]^c^	1.49 [1.08, 2.08]	1.11 [0.88, 1.73]	0.10
Inflammatory markers^‡^			
IL-1β, median [IQR]	0.05 [0.04, 0.10]	0.07 [0.05, 0.11]	0.989
TNF-α, median [IQR]	2.80 [2.23, 3.70]	3.07 [2.04, 3.62]	0.996
IL-6, median [IQR]	0.99 [0.57, 1.63]	1.07 [0.86, 1.26]	0.192
IL-8, median [IQR]	1.08 [0.95, 1.27]	1.12 [1.04, 1.31]	0.047
INF-γ, median [IQR]	3.19 [2.47, 5.34]	4.80 [3.73, 6.18]	0.241
IL-10, median [IQR]	0.28 [0.19, 0.45]	0.35 [0.26, 0.40]	0.977
IL12p70, median [IQR]	0.14 [0.09, 0.22]	0.16 [0.09, 0.22]	0.974
TGF-β, median [IQR]	3.04 [2.87, 3.26]	4.21 [3.98, 4.36]	0.022
CRP, median [IQR]	6.77 [6.38, 7.57]	6.37 [5.87, 6.99]	0.763
Education level			<0.001
Primary	56 (11.2%)	39 (27.3%)	
Secondary	86 (17.2%)	24 (16.8%)	
Technical	147 (29.4%)	49 (34.3%)	
Higher	211 (42.2%)	31 (21.7%)	
Social class, n (%)			0.022
Low	141 (28.2%)	54 (37.8%)	
Middle	139 (27.8%)	26 (18.2%)	
High	220 (44%)	63 (44.1%)	
Alcohol intake, n (%)			0.088
None	210 (42%)	54 (37.8%)	
1–128 mL/week	129 (25.8%)	28 (19.6%)	
129–265 mL/week	80 (16%)	29 (20.3%)	
266–461 mL/week	57 (11.4%)	18 (12.6%)	
≥ 462 mL/week	24 (4.8%)	14 (9.8%)	
Smoking status, n (%)			0.052
Never	265 (53%)	62 (43.4%)	
Smoked other than cigarettes	39 (7.8%)	17 (11.9%)	
Smoked < 15 pack years	28 (5.6%)	6 (4.2%)	
Smoked ≥ 15 but < 30 pack years	72 (14.4%)	19 (13.3%)	
Smoked ≥ 30 pack years	91 (18.2%)	38 (26.6%)	

^a^ Comparison of characteristics between men with dementia and/or MCI and those without at PRIME-COG follow up. Characteristics were compared using chi-squared tests for categorical variables and t-tests for continuous variables.

^b^ p value < 0.05 considered significant.

^c^ Data for blood inflammatory markers and blood triglycerides were not normally distributed, therefore, log transformed values were used for analysis. For descriptive purposes, median and interquartile ranges are presented.

### Periodontitis, dementia/MCI, and cognitive decline

Logistic regression models revealed no significant associations between periodontitis and incidence of dementia or MCI. In the sequential model design, moderate periodontitis did not display any further risk of dementia and/or MCI compared to mild or no periodontitis (fully adjusted model, OR 1.03, 95% CI 0.59, 1.77, p = 0.923). The OR for dementia and/or MCI in the presence of severe periodontitis was found to be lower compared to mild or no sign of periodontitis (fully adjusted model, OR 0.83, 95% CI 0.46, 1.45, p = 0.552) (Table 2), although this was not significant. In sensitivity analyses, we did not find any significant associations when testing for a trend across classification of periodontitis (‘severe’, ‘moderate’, ‘mild or no disease’), or when replacing periodontitis in the model with a differential classification of periodontal disease (mean clinical attachment loss measured in mm) (data not shown). Despite a lack of relationship between past periodontal disease, the fully adjusted logistic regression model identified number of teeth to display a significant inverse association with dementia and/or MCI (p = 0.023).

**Table 2.** Associations between periodontitis at PRIME rescreening and incident dementia and/or MCI at PRIME-COG follow up (n = 642)^1^.

Predictor variable	Model 1		Model 2		Model 3^a^		Model 4		Model 5	
	OR (95% CI)	*p* ^b^	OR (95% CI)	*p* ^b^	OR (95% CI)	*p* ^b^	OR (95% CI)	*p* ^b^	OR (95% CI)	*p* ^b^
Periodontitis										
(Moderate versus Mild or None)	1.09 (0.69, 1.71)	0.698	1.04 (0.64, 1.69)	0.860	1.08 (0.63, 1,82)	0.776	1.01 (0.58, 1.72)	0.984	1.03 (0.59, 1.77)	0.923
(Severe versus Mild or None)	0.95 (0.58, 1.54)	0.849	0.84 (0.48, 1.41)	0.510	0.94 (0.52, 1.64)	0.825	0.82 (0.44, 1.45)	0.497	0.83 (0.45, 1.50)	0.552
Age (per year increase)			1.13 (1.06, 1.21)	<0.001	1.12 (1.05, 1.21)	0.001	1.12 (1.04, 1.20)	0.002	1.12 (1.04, 1.21)	0.002
Education										
(Primary versus Higher)			5.61 (3.08, 10.38)	<0.001	6.12 (3.24, 11.79)	<0.001	5.86 (3.09, 11.37)	<0.001	5.39 (2.78, 10.68)	<0.001
(Secondary versus Higher)			2.05 (1.08, 3.85)	0.027	2.24 (1.12, 4.40)	0.020	2.28 (1.13, 4.54)	0.019	2.32 (1.14, 4.67)	0.019
(Technical versus Higher)			2.67 (1.58, 4.59)	<0.001	3.04 (1.72, 5.47)	<0.001	3.04 (1.71, 5.52)	<0.001	2.91 (1.61, 5.34)	<0.001
Socioeconomic status										
(Low versus High)			0.96 (0.60, 1.53)	0.875	1.12 (0.67, 1.86)	0.663	1.13 (0.67, 1.88)	0.653	1.07 (0.63, 1.80)	0.809
(Medium versus High)			0.61 (0.35, 1.03)	0.072	0.65 (0.35, 1.16)	0.150	0.65 (0.35, 1.18)	0.165	0.70 (0.38, 1.28)	0.257
*APOE* ε4										
(2 versus 0 allele)					9.47 (2.37, 47.8)	0.002	10.14 (2.44, 52.84)	0.002	11.21 (2.68, 58.70)	0.002
(1 versus 0 allele)					1.81 (1.10, 2.95)	0.018	1.88 (1.14, 3.09)	0.013	1.84 (1.10, 3.04)	0.018
Cardiovascular disease (Yes versus No)					1.72 (1.00, 2.90)	0.046	1.65 (0.95, 2.84)	0.071	1.57 (0.89, 2.75)	0.115
Diabetes (Yes versus No)					0.56 (0.31, 1.05)	0.064	0.61 (0.33, 1.14)	0.114	0.61 (0.33, 1.15)	0.117
Hypertension (Yes versus No)							1.83 (1.16, 2.89)	0.009	1.77 (1.10, 2.82)	0.018
Cholesterol (per mmol/l increase)							0.99 (0.78, 1.24)	0.899	1.00 (0.79, 1.27)	0.986
Number of teeth (per tooth increase)							0.95 (0.92, 0.99)	0.023	0.95 (0.91, 0.99)	0.023
Smoking										
(≥ 30 pack years versus Never)									1.23 (0.67, 2.22)	0.504
(≥ 15 but < 30 pack years versus Never)									1.14 (0.54, 2.31)	0.715
(Smoked < 15 pack years Never)									0.87 (0.29, 2.31)	0.786
(Smoked other than cigarettes versus Never)									1.72 (0.80, 3.60)	0.154
Alcohol intake										
(≥ 462 mL/week versus None)									1.47 (0.56, 3.67)	0.422
(266–461 mL/week versus None)									0.81 (0.37, 1.70)	0.580
(129–265 mL/week versus None)									1.20 (0.64, 2.21)	0.569
(1–128 mL/week versus None)									0.86 (0.46, 1.55)	0.612

*Association between periodontal disease at PRIME rescreening and dementia and/or mild cognitive impairment (MCI) at follow up assessed using logistic regression models. Data presented as odds ratios (OR) and (95% confidence intervals). Models including additional predictor variables and were constructed sequentially. Model 1 included only periodontal disease as the predictor variable. Model 2 incorporated age, education level and socioeconomic status. Model 3, number of *APOE* ε4 alleles, cardiovascular disease, and diabetes. Model 4, hypertension, blood cholesterol and number of teeth. The final model incorporated smoking status and alcohol consumption and represented the fully adjusted model.

^a^ Model 3 indicated to exhibit lowest AIC.

^b^ p value < 0.05 considered significant.

There was no significant relationship with periodontitis and the secondary endpoint, cognitive decline, with moderate periodontitis showing a negative association with cognitive decline compared to mild or no diagnosis of periodontitis (fully adjusted model, OR 0.48, 95% CI 0.13, 1.46, p = 0.234), while presence of severe periodontitis carried a positive association with cognitive decline (fully adjusted model, OR 1.34, 95% CI 0.48, 3.46, p = 0.562) (Supplemental Table 1), although neither were significant.

In the fully adjusted model, significant predictor variables associated with both dementia and/or MCI and cognitive decline included age (p = 0.002), all levels of education (primary, p < 0.001; secondary, p = 0.019; technical, p < 0.001), number of *APOE* ε4 risk alleles (1 versus 0, p = 0.018; 2 versus 0, p = 0.002) and hypertension (p = 0.018). Incident cardiovascular disease was significantly associated in model 3 (p = 0.046) (Table 2). In the models for cognitive decline, significantly associated variables included the number of *APOE* ε4 risk alleles (fully adjusted model, 1 versus 0, p = 0.012; 2 versus 0, p < 0.001), and primary versus higher education in models 2-4 (model 4, p = 0.030) (Supplemental Table 1).

### Serum levels of IgG antibodies to four common dental bacterial pathogens

**Figure 1.**
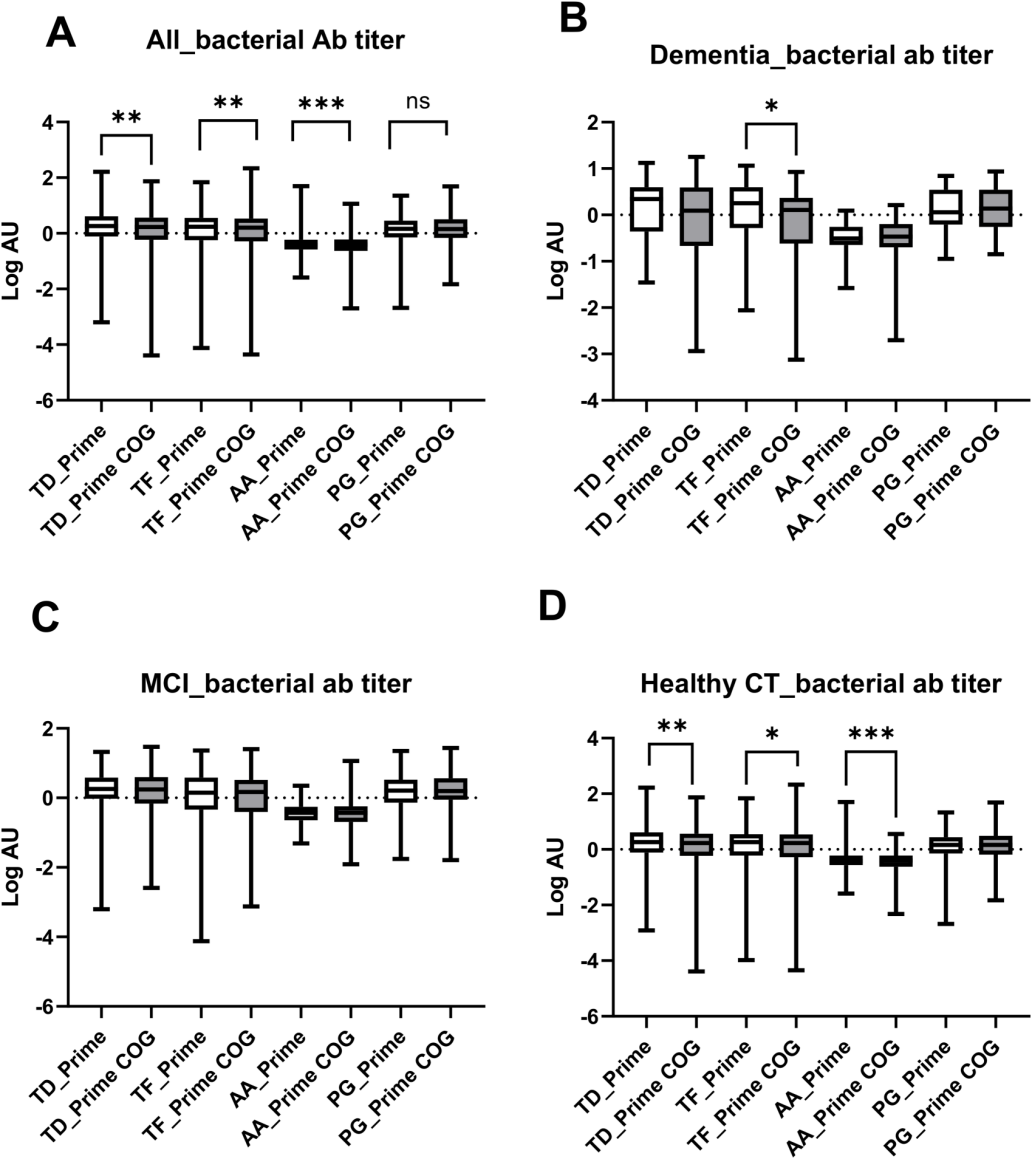
Serum IgG levels to *Treponema denticola* (*Td*), *Tannerella forsythia* (*Tf* ), *Aggregatibacter actinomycetemcomitans* (*Aa*), and *Porphyromonas gingivalis* (*Pg*) in all participants combined (A) and stratified into patients with Dementia (B), MCI (C), and healthy control individuals (D) at baseline (open bars, PRIME) and follow-up visit (closed bars, PRIME-COG).

**Figure 2.**
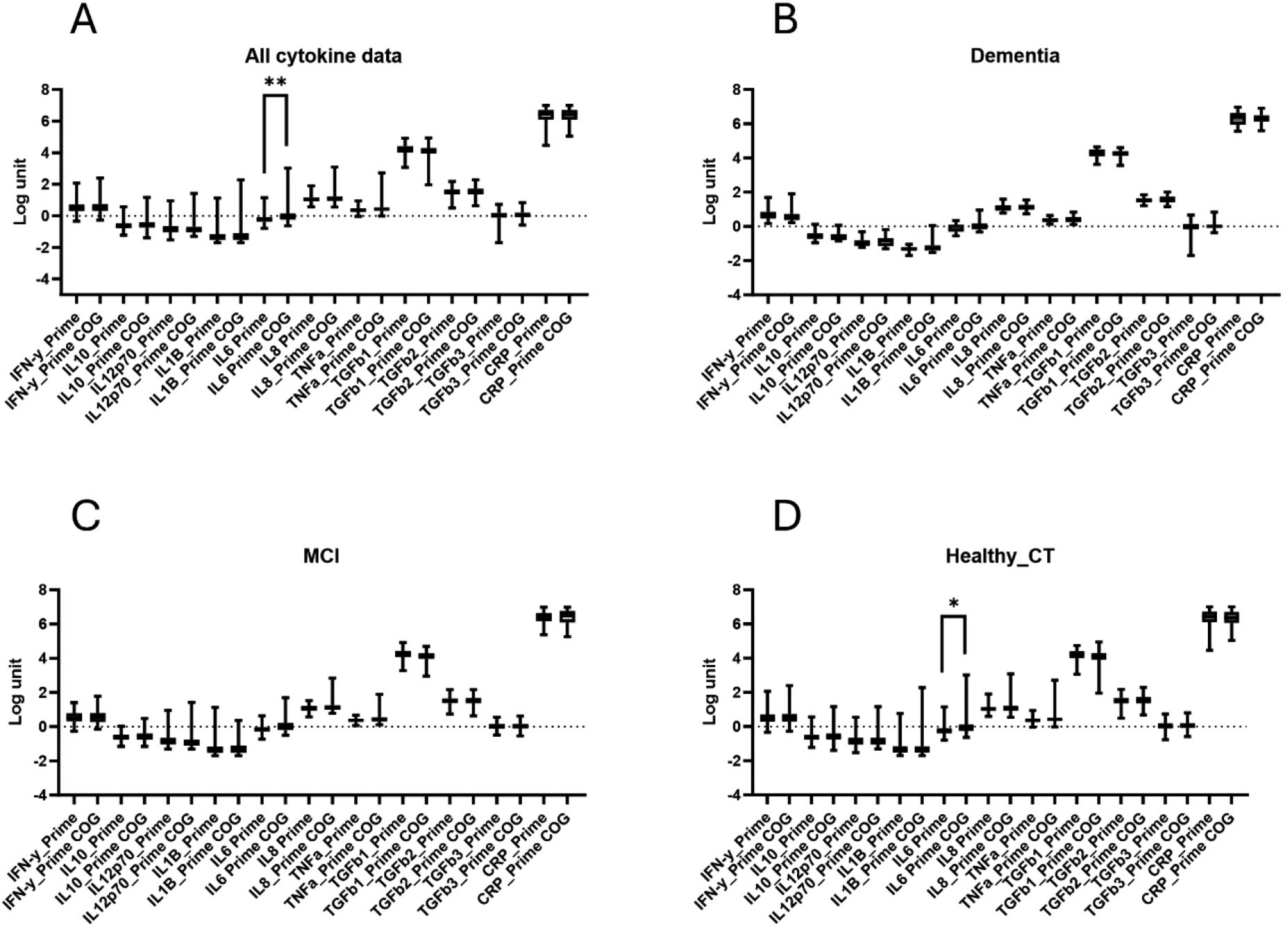
Serum levels of pro-inflammatory cytokines, TGF-β, and CRP at baseline and at follow-up visits in patients with Dementia, MCI, or healthy control participants. Serum levels of inflammatory markers in all participants combined (A) and stratified into patients with Dementia (B), MCI (C), and healthy control individuals (D) at baseline (PRIME) and follow-up visit (PRIME-COG). Analysis performed with log transformed values.

**Figure 3.**
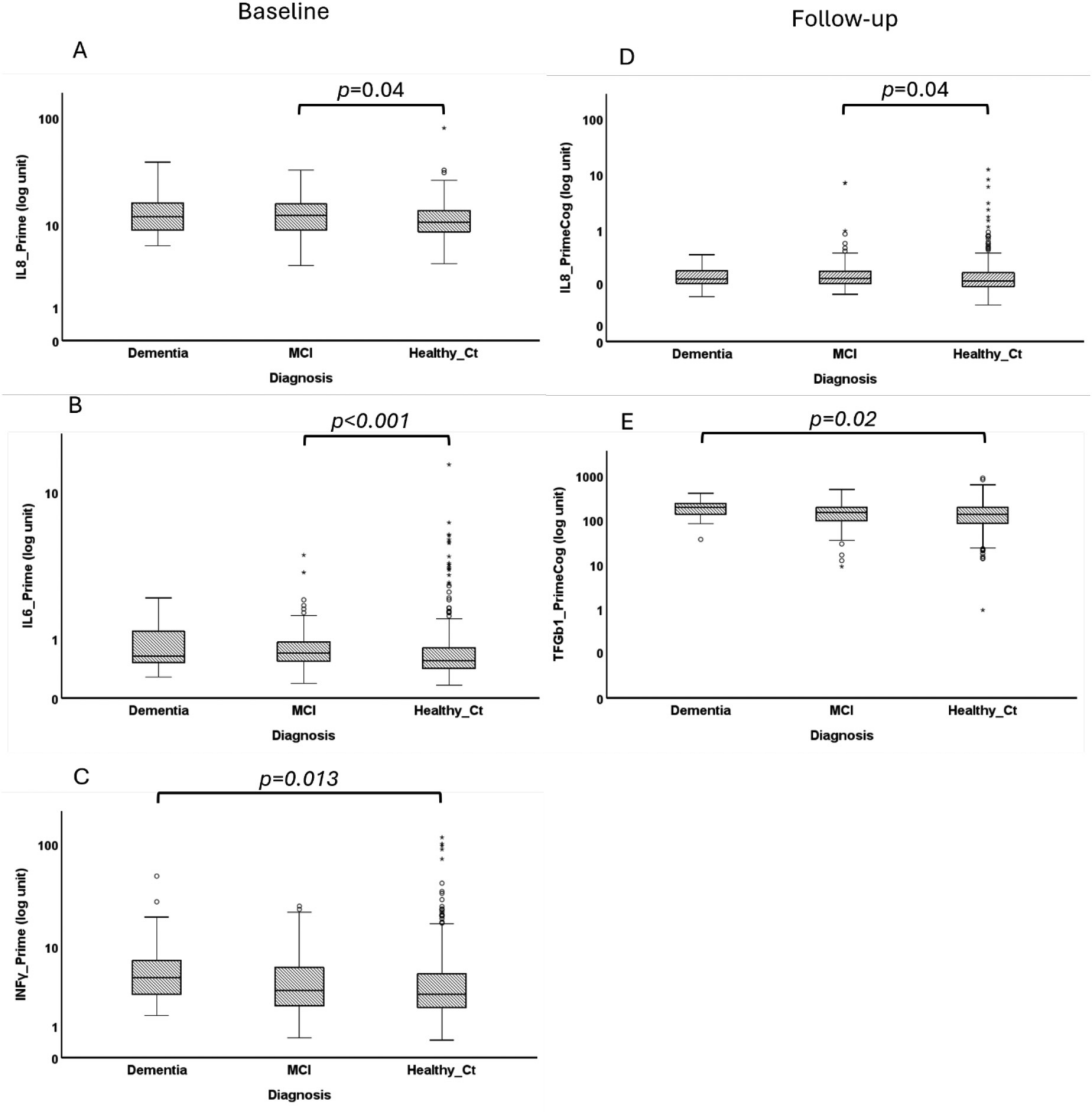
Comparison of serum levels of IL-8, IL-6, TGF-β, and INFγ at baseline (A–C) and follow-up (D–E) visits between patients with dementia or MCI to healthy control participants. Analysis performed with log transformed values.

Combined data of all participants indicated an overall decrease in the serum levels of IgG antibodies to *Td* (p = 0.099), *Tf* (p = 0.035)*, Aa* (p *=* 0.0007), and *Pg* (p = 0.615) over the 15-year follow-up period (Figure 1A). Stratification of participants by clinical group (i.e., dementia, MCI, cognitive normal/healthy) identified decreased serum IgG levels, over the 15–20-year follow-up period only in participants without cognitive decline (Figure 1D). In contrast, IgG levels to *Td, Tf,* and *Aa* remained constant over the study period in participants diagnosed with MCI (Figures 2 and 3) and dementia (Figure 3B), with the exception anti-*Tf* IgG, which decreased (p = 0.04). There was no significant difference in IgG antibodies levels for the four bacterial pathogens across the study groups (Supplemental Figure 1).

### Serum levels of pro-inflammatory cytokines, CRP, and TGF-β

Comparison of serum levels of pro-inflammatory cytokines, CRP and TGF-β at baseline and follow-up visits were not significantly different between participants with dementia, MCI, or without cognitive impairment (Figure 2). Stratifying participants by clinical groups revealed that men with MCI had significantly higher serum IL-8 levels at baseline (p = 0.04; Figure 3A) and follow-up (p = 0.04; Figure 3D) and elevated levels of IL-6 at baseline (p < 0.001; Figure 3B). Men that developed dementia showed elevated levels of IFN-γ at baseline (p = 0.013; Figure 3C) and TGF-β1 at follow-up (p = 0.02; Figure 3B) compared to cognitive healthy men.

**Figure 4.**
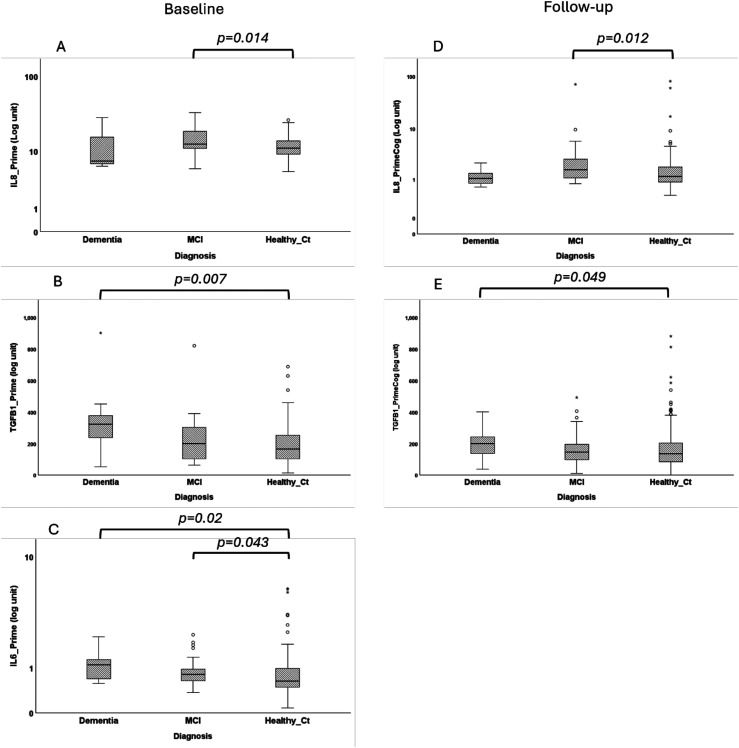
Serum levels of IL-8, TGF-β, and IL-6 at baseline (A–C) and at follow-up (D–E) visits in participants aged over 81 years old at follow up, diagnosed with MCI ordementia compared to healthy cognitive normal participants of the same age. Analysis performed with log transformed values.

To control for age, we next compared serum levels of pro-inflammatory cytokines in older participants only (>81 years old at follow-up). Participants with MCI had significantly higher serum levels of IL-8 at baseline (p = 0.014; Figure 4A) and follow-up (p = 0.012; Figure 4D) and elevated levels of IL-6 at baseline (p = 0.043; Figure 3C). Older participants that developed dementia showed elevated levels of TGF-β1 at baseline (p = 0.007; Figure 3B) and follow-up (p = 0.049; Figure 3E) compared to cognitive healthy men. Older participants that developed dementia also showed significant higher IL-6 levels at baseline (p = 0.02; Figure 3C), compared to cognitive healthy men

**Figure 5.**
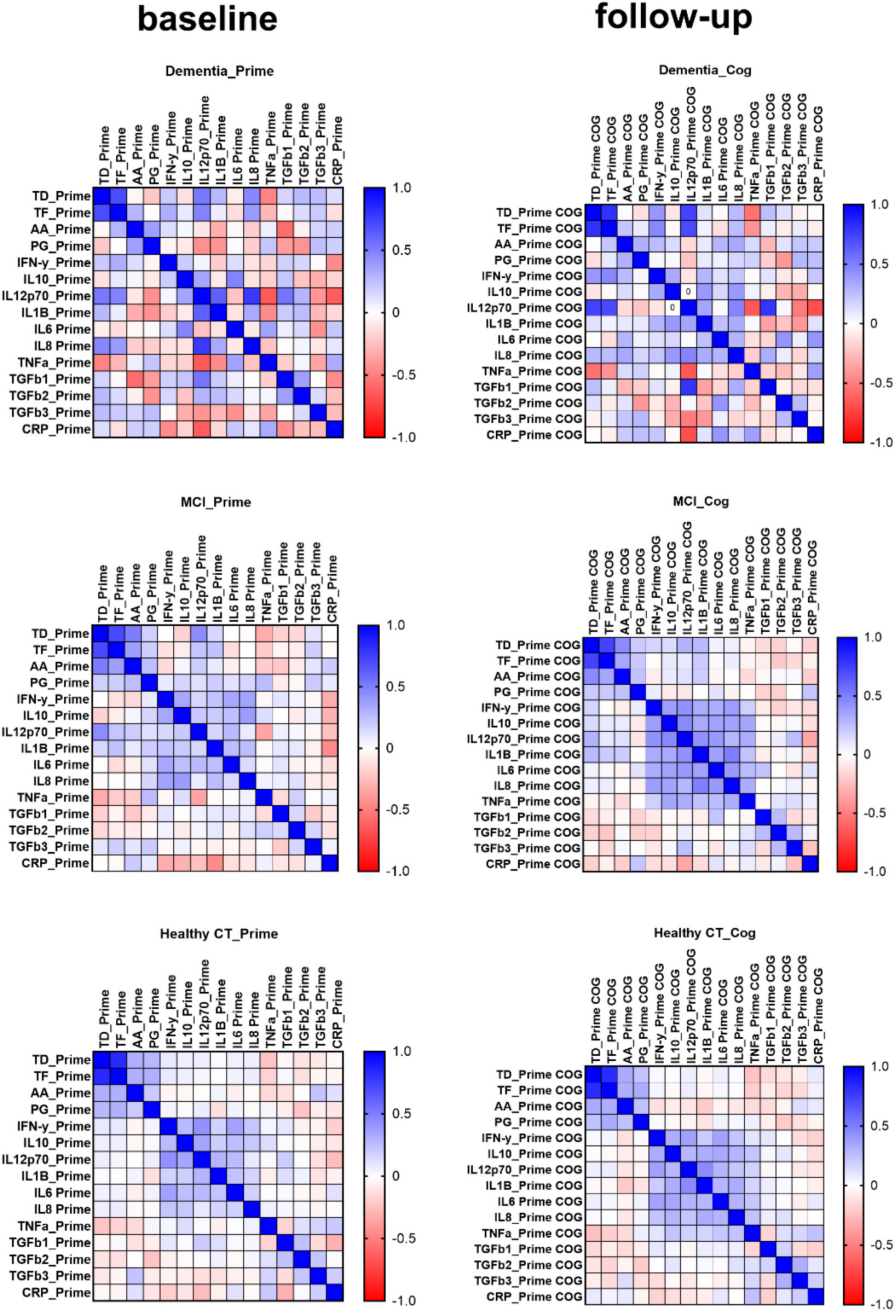
Correlation matrix of serum levels of IgG antibodies to common dentalbacteria, pro-inflammatory cytokines, TGF-β, and CRP in patients with Dementia, MCI, or in healthy control individuals at baseline (PRIME) and follow-up visit (PRIME-COG).

We next assessed if higher levels of serum inflammatory markers were associated with serum IgG antibodies to four common bacterial periodontal pathogens. A correlation matrix between serum inflammatory markers and IgG antibodies levels identified variation between participants with MCI and/or dementia to those who were cognitive healthy (Figure 5). Participants that developed dementia over the study period showed a positive correlation between baseline serum levels of IL-12p70 (r = 0.523, r = 0.486), and IL-8 (r = 0.468, r = 0.389) and IgG serum levels to the periodontal pathogens *Td* and *Tf,* respectively (Supplemental Figure 2). These participants also showed a negative correlation with IL-12p70 (r = −0.437), IL-1β (r = −0.422), TGFβ1 (r = −0.391), and TGFβ2 (r = −0.424) and serum IgG levels of *Pg* (Figure 5 and Supplemental Figure 2). These associations between serum inflammatory markers and IgG levels of periodontal pathogens were persistent over the follow-up period (Figure 5). Participants that developed MCI during the follow-up period showed positive correlations between serum levels of IL-8 and IgG to periodontal pathogens (r = 0.88 for *Td*; r = 0.98 for *Tf* and r = 0.92 for *Aa*) and CRP (r = 0.96 for *Td*; r = 0.82 for *Tf*), which were not observed in participants that remained cognitive healthy (Figure 5 and Supplemental Figure 2).

## Discussion

The main findings from the present study are that in older men residing in the Northern Ireland, a clinical diagnosis of periodontitis does not predict incident dementia, MCI or cognitive decline over a fifteen-year follow-up period. However, in men with a higher number of teeth at baseline, the risk of dementia and/or MCI was reduced, suggesting that maintaining adequate oral health and mitigating tooth loss may be a factor in reducing risk of cognitive decline in aging. Further, in men with dementia and/or MCI, blood levels of a number of inflammatory markers were higher compared to men with no cognitive impairment, highlighting the inflammatory component of perturbations in cognitive function. Serum levels of IL-6, IL-8, and IFN-γ were significantly elevated at baseline in men that developed MCI and/or dementia 15 years later, compared to men that remained cognitively healthy. The positive correlation between serum IgG levels to *Td* and *Tf* and inflammatory markers in participants that developed dementia and/or MCI may suggest an early and sustained immune response to common periodontal pathogens in men that develop MCI and/or dementia.

The lack of association between periodontitis and cognitive outcomes was somewhat unexpected, due to previous literature identifying significant longitudinal associations.^25,55^ However, it should be noted that this has not been consistently found, with other studies reporting a lack of significant longitudinal risk between oral health and dementia.^56,57^ Plausible reasons for the lack of significant associations in the present analysis could be due to study limitations: power, with low numbers of participants with dementia (4.8%), MCI (17.4%), and cognitive decline (5.8%). In addition, we did not carry out a periodontal examination at follow-up visits so we cannot rule out that disease improved for some men and was merely a transient occurrence. Oral status can change over the course of time, particularly when considering the ∼15 year follow up duration, therefore capturing tooth count at a single time point may bias results by not providing the entire picture for the relationship between longitudinal risk between periodontal disease and cognitive function. In the future, it would be advantageous to address such a limitation through repeat oral examinations and linkage to dental records, which would allow a fully comprehensive picture of both periodontal disease status, as well as overall oral health. In addition, our study population comprised Western European men only. A recent analysis reported that in individuals aged 60 to 69 years, AD prevalence in females was 1.9 times greater than that in males,^58^ which could a suggest a lower incident in our male only participants. The reasoning for the choice of study population lies in the PRIME cohort, which recruited a male only study cohort, in France and Northern Ireland, with the Northern Ireland cohort captured in the present analysis. The location of the PRIME cohort in Northern Ireland facilitated the opportunity to apply a thorough examination included in the present analysis by way of comprehensive cognitive assessments alongside standardized dental examinations performed by dental experts. It would be pertinent to build on the present findings in study populations of greater variety in both gender and ethnicity, and we are presently exploring such avenues. As is common among observational studies, the possibility of residual confounding or failure to account for other relevant confounders may have had some influence on the associations between periodontal disease and cognitive outcomes. Attrition bias should also be considered, participants who were not mobile and/or cognitively deteriorated may have been unlikely to have participated (including men who were deceased) in the PRIME COG follow-up, which therefore may have weakened any potential associations.

Despite a lack of relationship between periodontitis, our study identified a significant inverse association between number of teeth and future development of dementia and/or MCI. These observations agree with other longitudinal studies.^30^ Severe periodontitis is characterized by extensive bone loss, making teeth loose and prone to fall out. That being said, we did not find a difference of note between clinical attachment loss; a predominant clinical manifestation and determinant of periodontal disease, between men who developed cognitive impairment (mean 1.96 mm, SD 0.57) and those who did not (mean 2.24 mm, SD 0.87). While tooth loss due to periodontal destruction is a good indicator of previous susceptibility to periodontitis,^59^ other causes of tooth loss cannot be ruled out. Another factor is that this metric again was captured at the same time point as number of teeth and was not followed up, which as previously noted, would be enlightening to include in future investigations. A recent systematic review reported that the available evidence elucidating the relationship between number of teeth and dementia risk may be due to reverse causality, as well as exhibiting heterogeneity and lacking robust methodology to draw firm conclusions. Considering this, it may be that further well-designed studies involving standardized periodontal and cognitive assessments, as well as controlling for reverse causality are warranted. Our finding suggests that maintaining good oral health, and specifically, retention of teeth, may be a factor in reducing risk of cognitive impairment.

Our current study identified significant associations between MCI and dementia with known risk factors for dementia, including increasing age and carriage of *APOE* ε4 risk allele/s,^60^ lower education level and hypertension.^6^ Our results therefore help further contribute to the evidence base and establishment of risk factors for declines in cognitive function and dementia.

*T. denticola*, *T. forsythia*, and *A. actinomycetemcomitans* and *P. gingivalis*, are widely accepted as highly virulent periodontal pathogens, with other species identified as accessory pathogens.^61^ Multispecies microbial biofilms disrupt host tissue homeostasis and elicit a host immune-inflammatory response, resulting in expression of several cytokines and chemokines.^62^ Exposure to periodontal pathogens also promotes a humoral immune response and serum IgG antibody levels to periodontal pathogens have been reported to be associated with AD.^29^ In this study we found that serum IgG antibodies to periodontal pathogens remain constant during the study period in men that developed dementia and/or MCI, while a decrease was observed in cognitive healthy men. In men that developed dementia the serum IgG levels were associated with elevated levels of cytokines, and in particular IL12p70, IL-8, IL-6 and TGFβ. Men that developed MCI over the study period showed positive correlations between serum levels of IL-8 and IgG levels to periodontal pathogens. These observations highlight the potential role of systemic inflammation as a risk factor for dementia. The underlying mechanisms require further study, but may include a role for IL-8 mediated neutrophil function as reported previously.^63^

The strengths of the present study include one of the longest follow-up investigations into the longitudinal association between periodontal disease and dementia. The study utilized the PRIME cohort, representing a well phenotyped sample of older men and homogenous sample: white Western-European men, of a similar age, who at original recruitment were representative of the general population of Northern Ireland.^43^ The cognitive assessments were performed by experienced clinicians within our group who specialize in dementia/AD research, our analysis therefore benefitted from a consensus diagnosis of dementia, MCI and cognitive decline which was based on a comprehensive clinical cognitive assessment. The exposure variable was comprehensively assessed by a detailed full mouth periodontal examination, and the recommended case definitions of periodontitis for epidemiological research applied.^64^ The periodontal examination was also performed by a calibrated examiner rather than relying on self-report or insurance data. Further, the models implemented were appropriately adjusted for important confounders well known to increase risk of cognitive decline including *APOE* ε4, sociodemographic characteristics and risk factors for cardiometabolic disease, highlighted by the significant relationships identified in the analysis.

In conclusion, in a cohort of older men residing in Northern Ireland, periodontitis did not influence longitudinal risk of dementia or MCI following a fifteen-year follow-up; however, a greater number of teeth was associated with reduced longitudinal risk of dementia and/or MCI. In addition, circulating blood markers of inflammation were higher, and IgG titers to common periodontal pathogens remained constant in men diagnosed with dementia or MCI, while IgG titers significantly reduced over time in cognitive normal men. Our findings therefore help contribute to the evidence base on the longitudinal associations between periodontitis and dementia. Periodontitis is treatable, and therefore if identified as a predictor of dementia, represents a modifiable risk factor. Intervention studies are warranted to provide more clear evidence for periodontitis initiating MCI and/or dementia.

## Supplemental Material

sj-docx-1-alz-10.1177_13872877251401563 - Supplemental material for Periodontitis and incident cognitive decline and dementia: A 15-year prospective cohort study of older men residing in Northern Ireland Supplemental material, sj-docx-1-alz-10.1177_13872877251401563 for Periodontitis and incident cognitive decline and dementia: A 15-year prospective cohort study of older men residing in Northern Ireland by Dominic N Farsi, Rena Abadalkareem, Gerry J Linden, Gareth J McKay, Claire T McEvoy, Michael McAlinden, Lewis Winning, Michael Hurley, Jo Kelly, Peter A Passmore, Clive Holmes, Chris C Patterson, Jessica L Teeling and Bernadette McGuinness in Journal of Alzheimer's Disease
