# Supplementary material for: Periodontitis and incident cognitive decline and dementia: A 15-year prospective cohort study of older men residing in Northern Ireland
Source: J Alzheimers Dis. 2025 Dec 12;109(2):980–95. doi: 10.1177/13872877251401563 (PMC12775178; doi:10.1177/13872877251401563)
Supplement: sj-docx-1-alz-10.1177_13872877251401563 - Supplemental material for Periodontitis and incident cognitive decline and dementia: A 15-year prospective cohort study of older men residing in Northern Ireland [file sj-docx-1-alz-10.1177_13872877251401563.docx]

**Supplemental Material**

**Periodontitis and incident cognitive decline and dementia: A 15-year prospective cohort study of older men residing in Northern Ireland**

**Supplemental Methods**

*Determination of serum IgG levels to dental bacterial pathogens*

*Generating bacterial stocks for bacterial antigen.* *Tannerella forsythia* (*Tf*, FDC 338), *Treponema denticola* (*Td*), *A. actinomycetemcomitans* (*Aa*) and three strains of *P. gingivalis* (*Pg*), W83, 2561, RB22 D-1), were obtained from the American Type Culture Collection (ATCC). The periodontal bacterial strains were cultured according to the supplier’s instruction. Briefly, bacteria were grown on agar plates to pick single colonies, followed by culturing in liquid broth incubated at 37°C under anaerobic conditions, until late exponential phase. The cultures were centrifuged at 6,000 *g* for 30 min at 5°C, and the isolated bacterial pellet was suspended in PBS, fixed with formaldehyde and stored at 4°C.

*Periodontal IgG titers in serum.* 96-well plates (Maxisorb, Nunc for *Tf,* *Td* and *Pg*; Polysorb, Nunc for *Aa*) were coated with 100 μl fixed bacteria diluted 1:500 in 50 mM sodium carbonate coating buffer pH 9.6 and incubated overnight at 4°C. The bacterial antigens were fixed onto the plate with 0.05% (v/v) glutaraldehyde solution for 15 min at room temperature (RT). The plates were washed with 100 μl wash buffer (PBS with 0.05% (v/v) Tween 20) three times, followed by adding 100 μl blocking buffer (5% BSA (w/v) in 1x PBS), and incubation for 30 min at RT in a microplate shaker (600 prm). After three washes, 100 μl of serum samples diluted 1:1000 in blocking buffer and standards were incubated for 1 h at RT, while shaking at 600 rpm. The standards that were used are human intravenous IgG (Vigam Liquid, Bio product Laboratory). After three washes, IgG binding to bacterial antigens was detected by adding 100 μl rabbit anti-human IgG horse-radish peroxidase (HRP) conjugated antibody (Sigma Merck) at 1:20,000 dilution with blocking buffer. The plates were incubated for 45 min at RT. After washing excess detection antibodies, 100 μl of TMB substrate (Pierce) was added to the plates and incubated for up to 20 min. The reaction was stopped by adding 100 μl stop solution (1M Sulfuric acid). The reaction was read at 450 nm using a plate reader (Tecan Infinite, F200 Pro).

**Supplemental Table 1.** Associations between periodontitis at PRIME rescreening and incident cognitive decline at PRIME-COG follow up (n=642)^1^

| **Predictor variable** | | **Model 1** | | **Model 2** | | | **Model 3**^†^ | | | **Model 4** | | | **Model 5** | | | |
| --- | --- | --- | --- | --- | --- | --- | --- | --- | --- | --- | --- | --- | --- | --- | --- | --- |
|  | | OR  (95% CI) | *p*^‡^ | OR  (95% CI) | *p*^‡^ | OR  (95% CI) | | *p*^‡^ | OR  (95% CI) | | *p*^‡^ | OR  (95% CI) | | ^p‡^ |  |  |
| Periodontitis | |  |  |  |  |  | |  |  | |  |  | |  |  |  |
| (Moderate versus Mild or None) | | 0.50  (0.17, 1.23) | 0.167 | 0.52  (0.17, 1.32) | 0.200 | 0.45  (0.12, 1.31) | | 0.178 | 0.47  (0.13, 1.39) | | 0.211 | 0.48  (0.13, 1.46) | | 0.234 |  |  |
| (Severe versus Mild or None) | | 0.96  (0.40, 2.11) | 0.924 | 0.96  (0.38, 2.19) | 0.921 | 1.21  (0.45, 2.98) | | 0.686 | 1.25  (0.45, 3.18) | | 0.654 | 1.34  (0.48, 3.46) | | 0.562 |  |  |
| Age (per year increase) | |  |  | 1.07  (0.95, 1.19) | 0.250 | 1.09  (0.96, 1.24) | | 0.168 | 1.10  (0.96, 1.25) | | 0.163 | 1.11  (0.97, 1.27) | | 0.125 |  |  |
| Education | |  |  |  |  |  | |  |  | |  |  | |  |  |  |
| (Primary versus Higher) |  |  | 3.20  (1.35, 7.58) | **0.008** | 2.92  (1.14, 7.54) | | **0.025** | 2.85  (1.10, 7.40) | | **0.030** | 2.23  (0.80, 6.18) | | 0.122 |  |  |  |
| (Secondary versus Higher) |  |  | 0.52  (0.12, 1.64) | 0.311 | 0.67  (0.15, 2.23) | | 0.545 | 0.68  (0.15, 2.28) | | 0.566 | 0.61  (0.13, 2.10) | | 0.468 |  |  |  |
| (Technical versus Higher) |  |  | 0.48  (0.15, 1.32) | 0.178 | 0.54  (0.15, 1.61) | | 0.292 | 0.54  (0.15, 1.65) | | 0.308 | 0.49  (0.13, 1.54) | | 0.247 |  |  |  |
| Socioeconomic status | |  |  |  |  |  | |  |  | |  |  | |  |  |  |
| (Low versus High) |  |  | 0.44  (0.17, 1.06) | 0.079 | 0.44  (0.15, 1.14) | | 0.112 | 0.45  (0.15, 1.19) | | 0.126 | 0.39  (0.13, 1.06) | | 0.079 |  |  |  |
| (Medium versus High) |  |  | 0.59  (0.22, 1.41) | 0.260 | 0.52  (0.17, 1.37) | | 0.210 | 0.54  (0.18, 1.43) | | 0.243 | 0.51  (0.17, 1.35) | | 0.198 |  |  |  |
| *APOE* ε4 | |  |  |  |  |  | |  |  | |  |  | |  |  |  |
| (2 versus 0 allele) |  |  |  |  | 21.38  (4.26, 53.90) | | **<0.001** | 23.47  (4.63, 55.46) | | **<0.001** | 30.15  (5.60, 60.54) | | **<0.001** |  |  |  |
| (1 versus 0 allele) |  |  |  |  | 2.82  (1.23, 6.39) | | **0.013** | 2.86  (1.25, 6.52) | | **0.012** | 2.89  (1.25, 6.68) | | **0.012** |  |  |  |
| Cardiovascular disease (Yes versus No) | |  |  |  |  | 1.67  (0.64, 4.01) | | 0.267 | 1.49  (0.56, 3.62) | | 0.399 | 1.47  (0.54, 3.65) | | 0.423 |  |  |
| Diabetes (Yes versus No) | |  |  |  |  | 0.72  (0.27, 2.31) | | 0.542 | 0.74  (0.27, 2.40) | | 0.585 | 0.74  (0.26, 2.48) | | 0.591 |  |  |
| Hypertension (Yes versus No) | |  |  |  |  |  | |  | 1.56  (0.68, 3.46) | | 0.278 | 1.31  (0.54, 3.08) | | 0.537 |  |  |
| Cholesterol (per mmol/l increase) | |  |  |  |  |  | |  | 0.84  (0.53, 1.30) | | 0.436 | 0.84  (0.53, 1.31) | | 0.458 |  |  |
| Number of teeth (per tooth increase) | |  |  |  |  |  | |  | 1.00  (0.93, 1.08) | | 0.952 | 1.00  (0.93, 1.08) | | 0.946 |  |  |
| Smoking | |  |  |  |  |  | |  |  | |  |  | |  |  |  |
| (≥ 30 pack years versus Never) |  |  |  |  |  | |  |  | |  | 1.51  (0.51, 4.32) | | 0.441 |  |  |  |
| (≥ 15 but < 30 pack years versus Never) |  |  |  |  |  | |  |  | |  | 1.08  (0.24, 3.90) | | 0.909 |  |  |  |
| (Smoked < 15 pack years Never) |  |  |  |  |  | |  |  | |  | 2.11  (0.29, 9.90) | | 0.385 |  |  |  |
| (Smoked other than cigarettes versus Never) |  |  |  |  |  | |  |  | |  | 2.30  (0.57, 7.84) | | 0.201 |  |  |  |
| Alcohol intake | |  |  |  |  |  | |  |  | |  |  | |  |  |  |
| (≥ 462 mL/week versus None) |  |  |  |  |  | |  |  | |  | 2.18  (0.45, 9.26) | | 0.305 |  |  |  |
| (266–461 mL/week versus None) |  |  |  |  |  | |  |  | |  | 1.06  (0.23, 4.09) | | 0.940 |  |  |  |
| (129–265 mL/week versus None) |  |  |  |  |  | |  |  | |  | 0.90  (0.25, 2.84) | | 0.864 |  |  |  |
| (1–128 mL/week versus None) |  |  |  |  |  | |  |  | |  | 1.19  (0.40, 3.37) | | 0.749 |  |  |  |
| ^*^Association between periodontal disease at PRIME rescreening and cognitive decline (a ≥ 3 point drop in MMSE score from PRIME rescreening to follow up) at follow up assessed using logistic regression models. Data presented as odds ratios (OR) and (95% confidence intervals). Models including additional predictor variables and were constructed sequentially. Model 1 included only periodontal disease as the predictor variable. Model 2 incorporated age, education level and socioeconomic status. Model 3, number of APOE-ε4 alleles, cardiovascular disease, and diabetes. Model 4, hypertension, blood cholesterol and number of teeth. The final model incorporated smoking status and alcohol consumption and represented the fully adjusted model.  ^†^Model 3 indicated to exhibit lowest AIC ^‡^p value < 0.05 considered significant. | | | | | | | | | | | | | | |  |  |

**Supplemental Figure 1.** Serum IgG levels to Td, Tf, Aa, and Pg at baseline and follow-up in patients with dementia, MCI or in control individuals

***
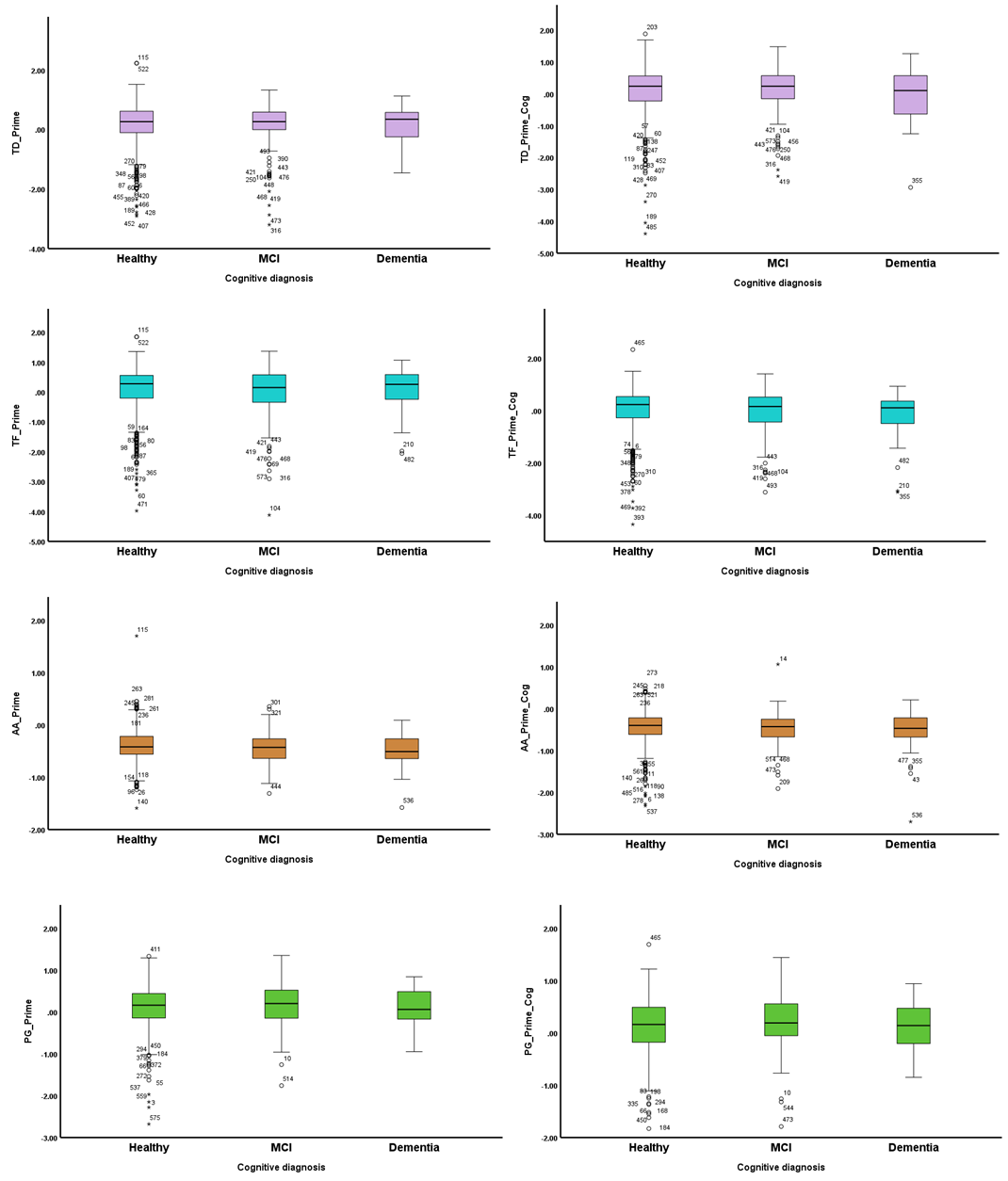
***

**Supplemental Figure 2.** Correlation coefficients of serum levels of IgG antibodies to common dental bacteria (Tg, TF, AA, and Pg), pro-inflammatory cytokines, TGF-β and CRP in patients with Dementia, MCI or in healthy control individuals at baseline (PRIME) and follow-up visit (PRIME-COG)
